# Supplementary material for: The interactome of intact mitochondria by cross-linking mass spectrometry provides evidence for coexisting respiratory supercomplexes
Source: Mol Cell Proteomics. 2017 Dec 8;17(2):216–32. doi: 10.1074/mcp.RA117.000470 (PMC5795388; doi:10.1074/mcp.RA117.000470)
Supplement: Supplemental Data [file supp_RA117.000470_133922_0_supp_23978_fzffwf.zip › spectra_annotation/mito_DR_spectra_annotation/307-1-4-1-4-1.pdf]

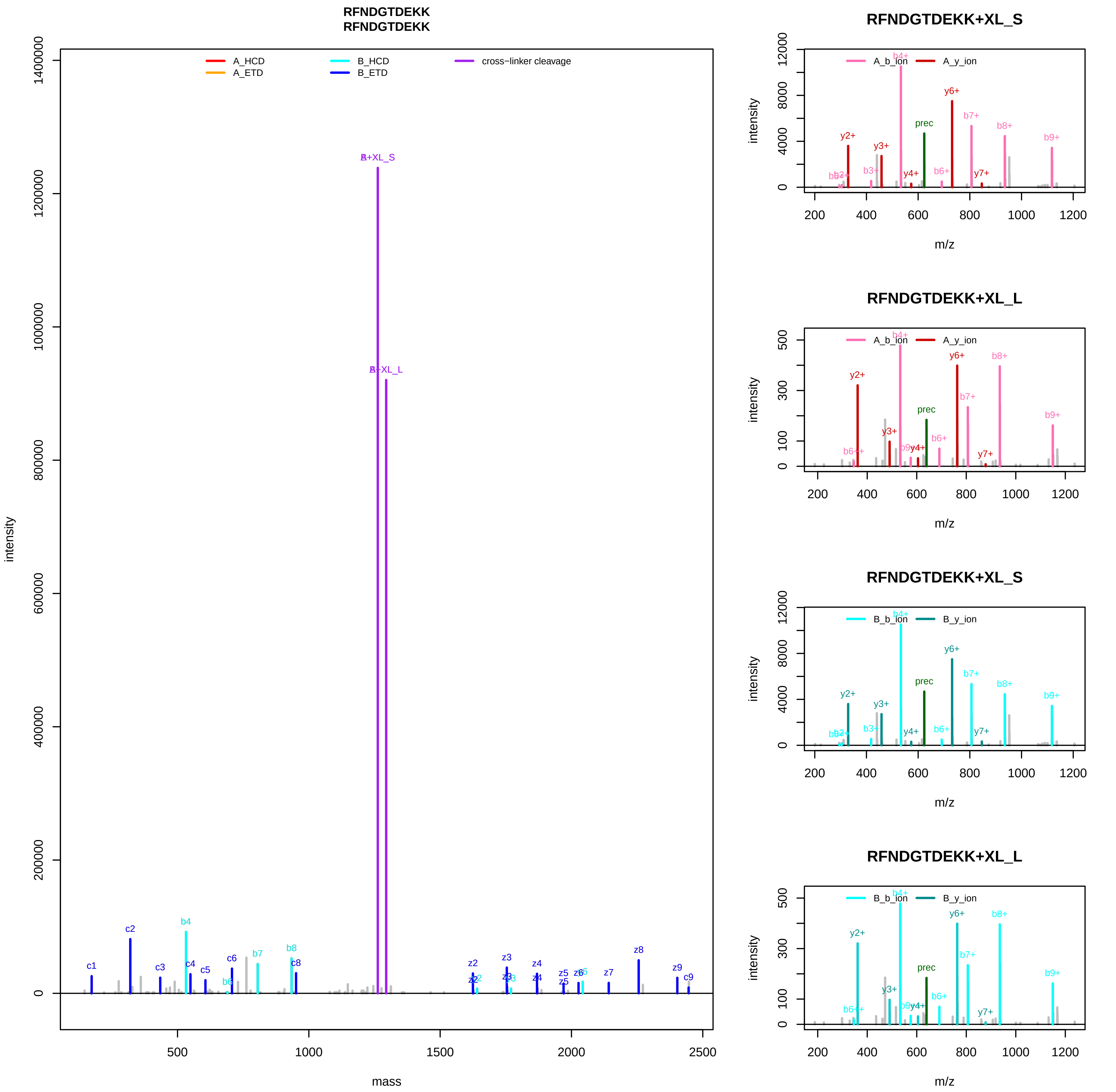
RFNDGTDEKK
RFNDGTDEKK
A_HCD
A_ETD
B_HCD
B_ETD
cross−linker cleavage
B+XL_S
B+XL_L
c1
c2
c3
b4
c4
c5
c6
b6
b7
b8
c8
z2
z3
z4
z5
z6
z7
z8
z9
c9
intensity
mass
0
200000
400000
600000
800000
1000000
1200000
1400000
500
1000
1500
2000
2500
RFNDGTDEKK+XL_S
A_b_ion
A_y_ion
b4+
y6+
prec
b7+
b8+
b9+
y2+
y3+
b3+
y4+
b6+
y7+
intensity
m/z
RFNDGTDEKK+XL_L
RFNDGTDEKK+XL_S
B_b_ion
B_y_ion
RFNDGTDEKK+XL_L
0
4000
8000
12000
0
100
300
500
200
400
600
800
1000
1200
